# Supplementary figures and images for: Thiocyanate and Organic Carbon Inputs Drive Convergent Selection for Specific Autotrophic Afipia and Thiobacillus Strains Within Complex Microbiomes
Source: Front Microbiol. 2021 Apr 8;12:643368. doi: 10.3389/fmicb.2021.643368 (PMC8061750; doi:10.3389/fmicb.2021.643368)

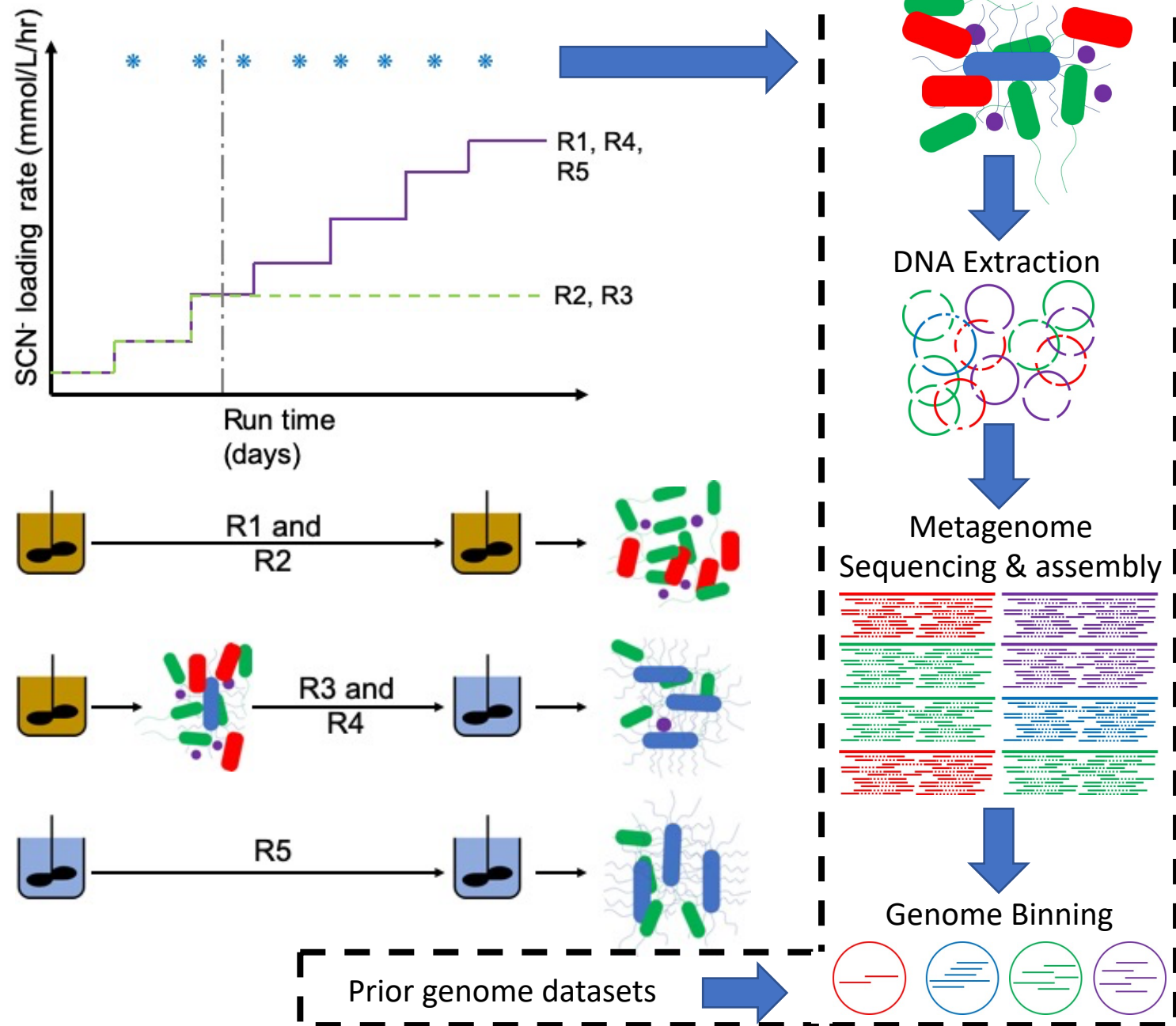

Supplement: Supplementary Figure 1 — Diagram summarizing the operation and sampling of the five SCN–reactors over the course of the experimental period. (A) shows the experimental SCN–feed loading regime over time, while (B) shows the five SCN–degrading reactors in operation. The vertical dotted lines and asterisks show the points at which biomass was sampled for subsequent total genomic DNA extractions and sequencing. [file Image_1.PDF]

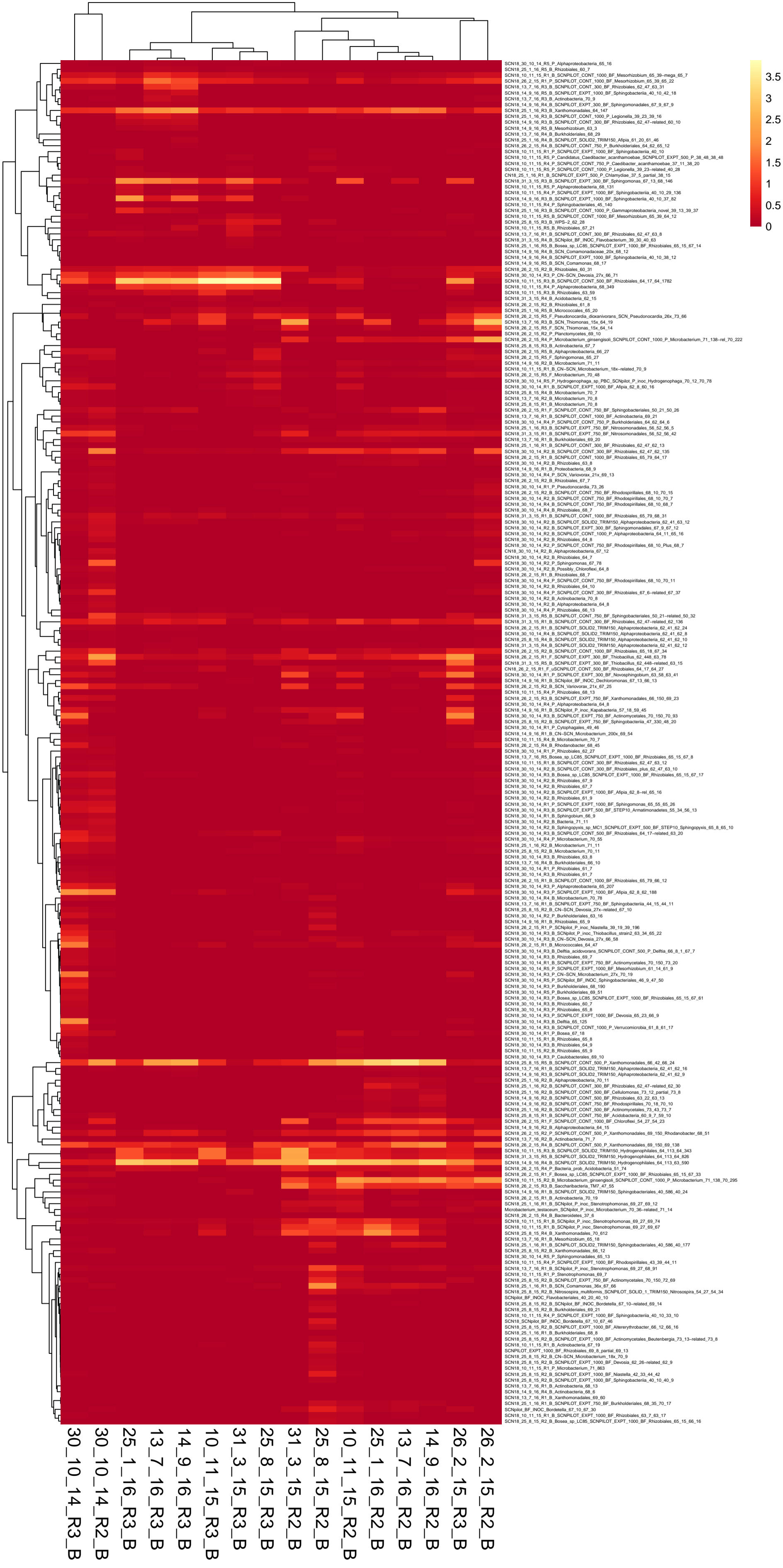

Supplement: Supplementary Figure 2 — Heatmap based on normalized relative abundance patterns of bacteria in R2 and R3 indicating co-occurrence of Saccharibacteria (TM7) and Microbacterium bins. [file Image_2.PDF]

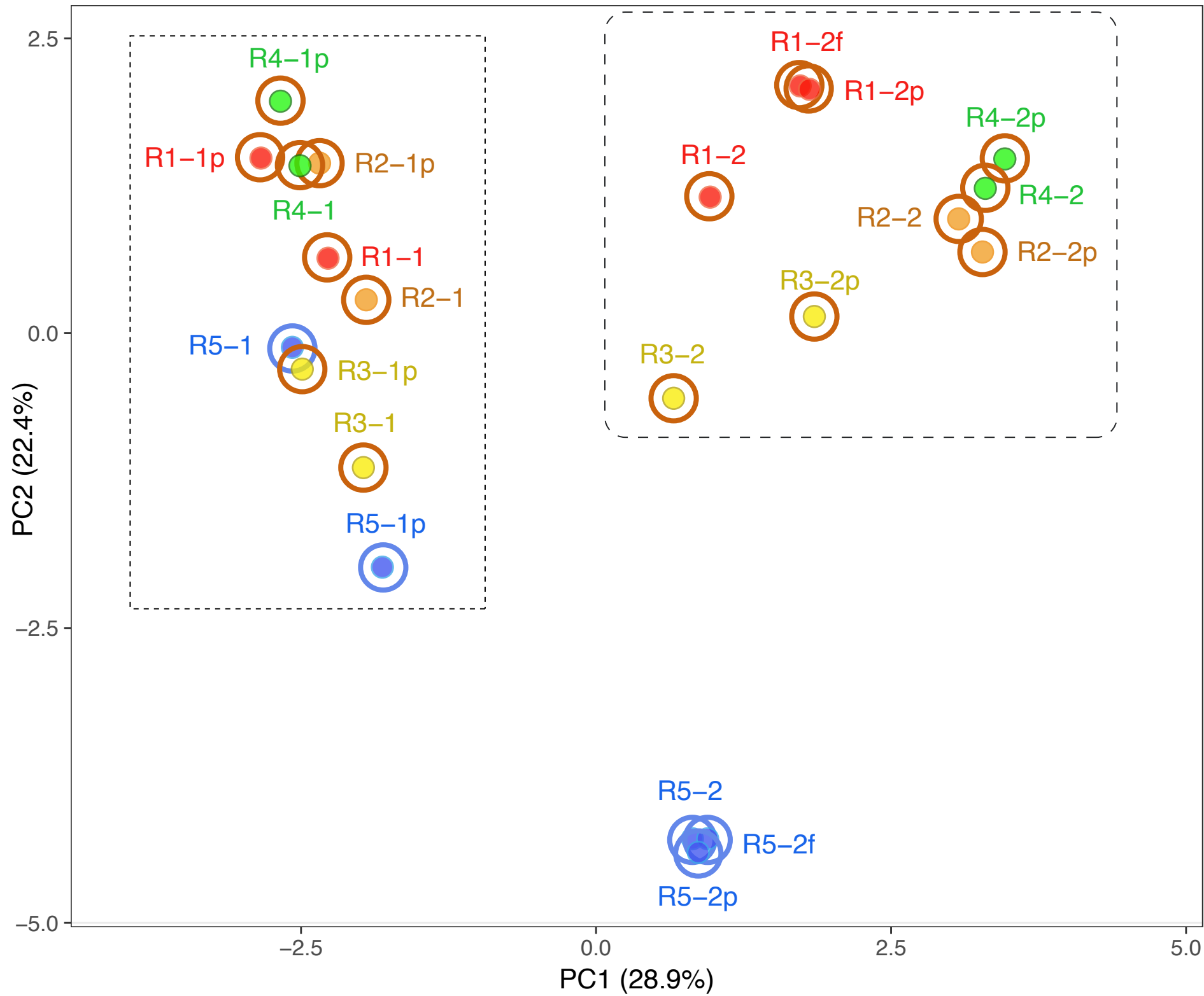

Supplement: Supplementary Figure 3 — Principal coordinate analysis of community composition at the first two time points in all reactors. The rectangular box shows that communities in all reactors were similar at the first time point, despite reactor 5 receiving no molasses for 117 days. The rounded box indicates communities in all reactors receiving molasses and 250 ppm SCN– (R1, R2, R3, and R4, the second time point). Despite experiencing the same treatments, R1, R2, R3, and R4 are somewhat distinct from each other. Reactor 5 (molasses-free reactor) after the second time point has a very different community composition compared to the other reactors. Included are samples from biofilm, the planktonic fraction (p) and, for reactors 1 and 5, floc samples (f). In most cases, the planktonic fraction is somewhat different from the biofilm (and floc samples). [file Image_3.PDF]

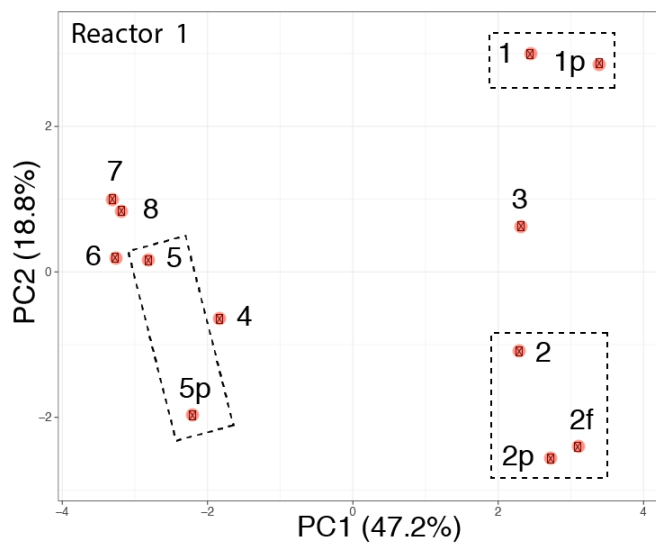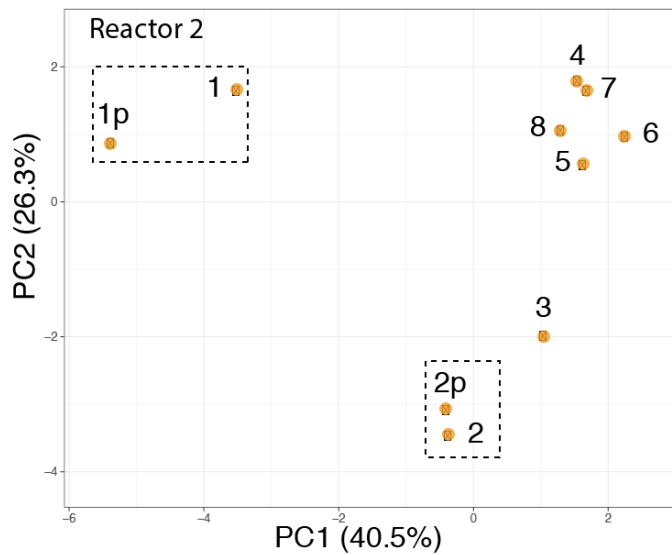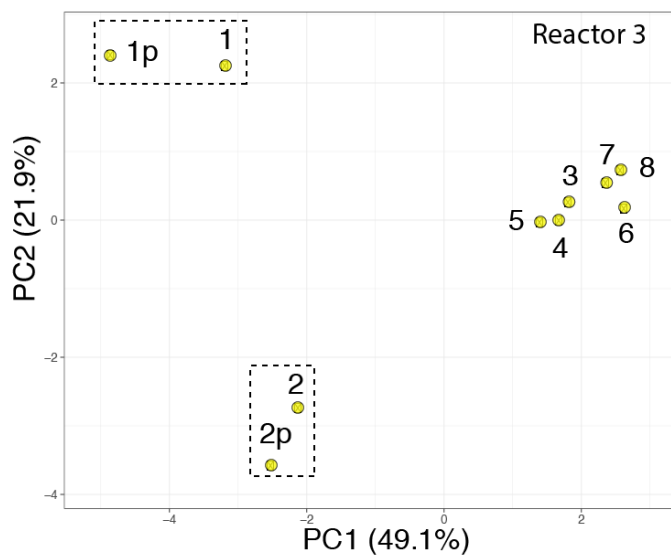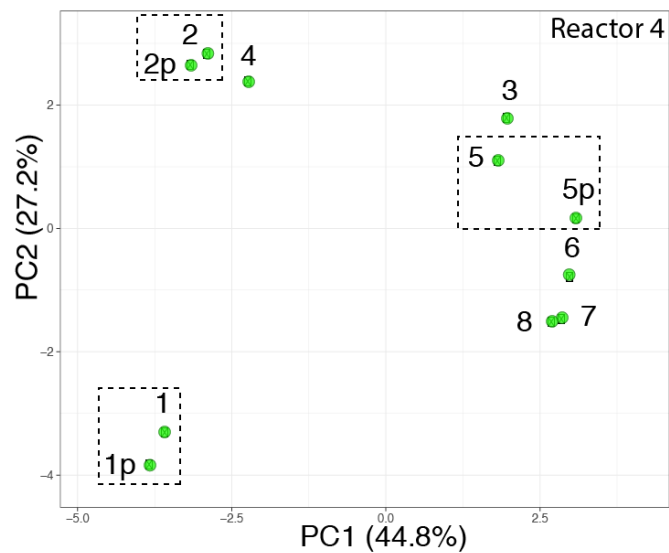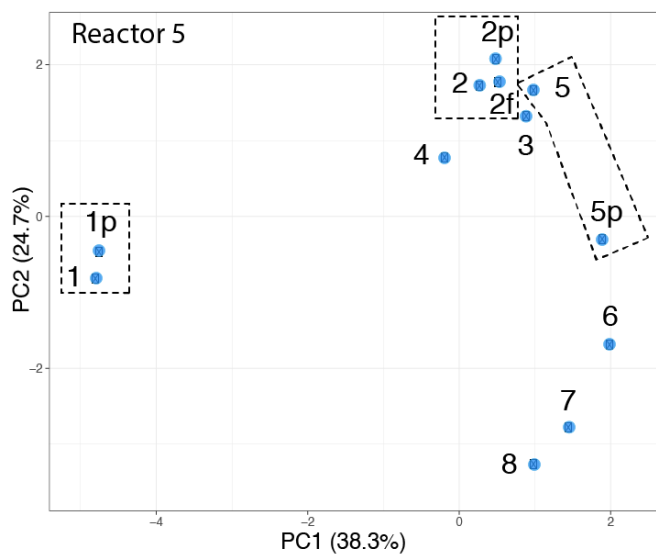

Supplement: Supplementary Figure 4 — Principal coordinate analysis of community composition for each of the five reactors. Boxes indicate biofilm, planktonic (p) and floc (f) samples from the same time point. [file Image_4.PDF]

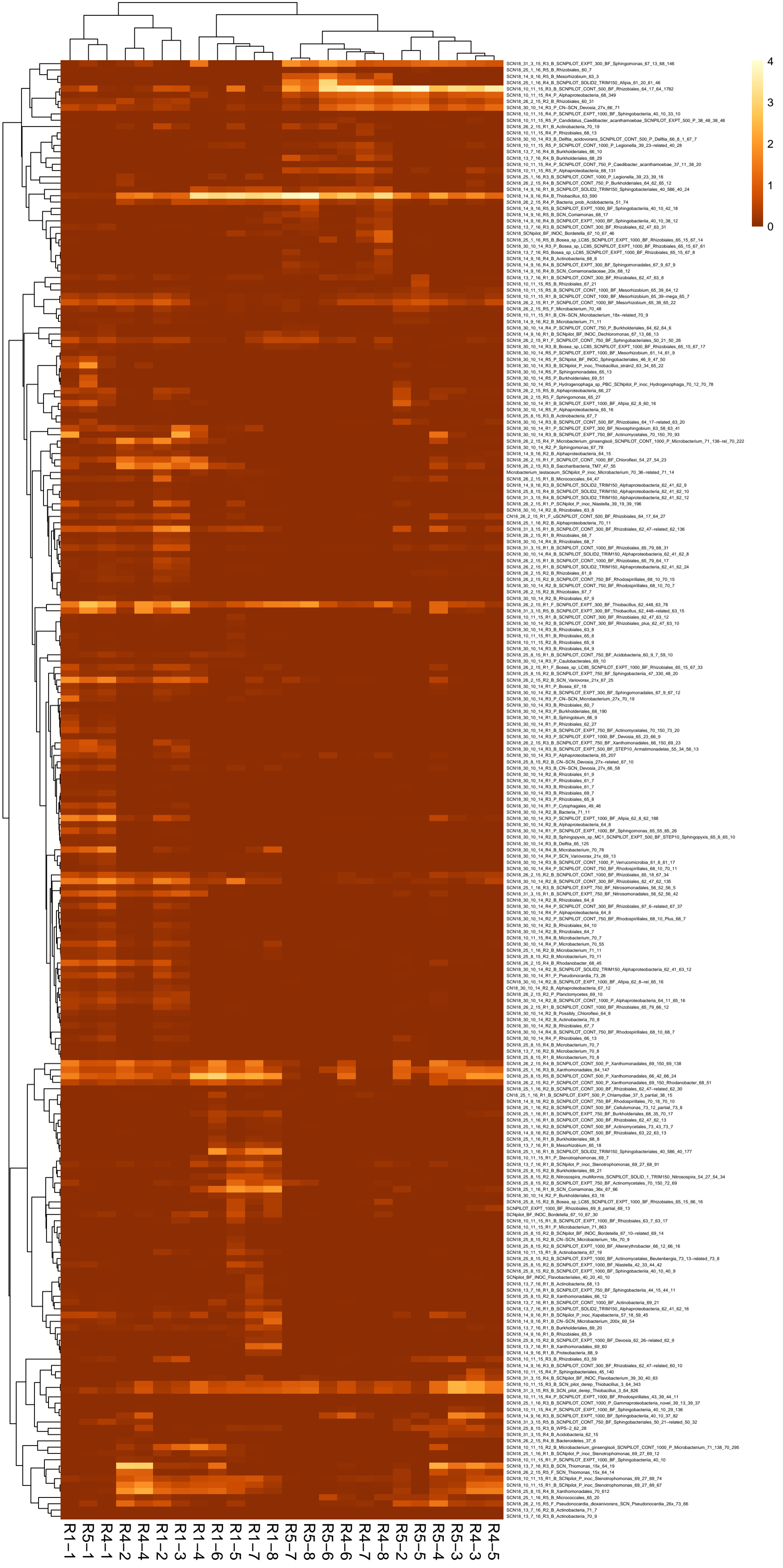

Supplement: Supplementary Figure 5 — Version of Figure 6B showing organism names. [file Image_5.PDF]
